# Performance Evaluation of Montelukast Pediatric Formulations: Part I—Age-Related *In Vitro* Conditions

**DOI:** 10.1208/s12248-021-00661-2

**Published:** 2022-01-10

**Authors:** Mariana Guimarães, Pascal Somville, Maria Vertzoni, Nikoletta Fotaki

**Affiliations:** 1 Department of Pharmacy and Pharmacology, University of Bath, Bath, UK; 2 UCB Pharma S.A., Product Development, B-1420 Braine l’Alleud, Belgium; 3 Department of Pharmacy, National and Kapodistrian University of Athens, Athens, Greece; 4 Centre for Therapeutic Innovation and Department of Pharmacy and Pharmacology, University of Bath, Claverton Down, Bath, BA2 7AY UK

**Keywords:** adults, age-related biorelevant dissolution, biopharmaceutics, montelukast, pediatrics

## Abstract

This study aimed to explore the potential of biopharmaceutics *in vitro* tools to predict drug product performance in the pediatric population. Biorelevant dissolution set-ups were used to predict how age and medicine administration practices affect the *in vitro* dissolution of oral formulations of a poorly water-soluble compound, montelukast. Biorelevant age-appropriate dissolution studies of Singulair^®^ (granules and chewable tablets) were conducted with the µDISS profiler™, USP 4 apparatus, USP 2 apparatus, and mini-paddle apparatus. Biorelevant simulating fluids representative of adult and pediatric conditions were used in the dissolution studies. The biorelevant dissolution conditions were appropriately selected (*i.e.* volumes, transit times, *etc.*) to mimic the gastrointestinal conditions of each of the subpopulations tested. Partial least squares regression (PLS-R) was performed to understand the impact of *in vitro* variables on the dissolution of montelukast. Montelukast dissolution was significantly affected by the *in vitro* hydrodynamics used to perform the dissolution tests (µDISS profiler™: positive effect); choice of simulation of gastric (negative effect) and/or intestinal conditions (positive effect) of the gastrointestinal tract; and simulation of prandial state (fasted state: negative effect, fed state: positive effect). Age-related biorelevant dissolution of Singulair^®^ granules predicted the *in vivo* effect of the co-administration of the formulation with applesauce and formula in infants. This study demonstrates that age-appropriate biorelevant dissolution testing can be a valuable tool for the assessment of drug performance in the pediatric population.

**Graphical Abstract:**

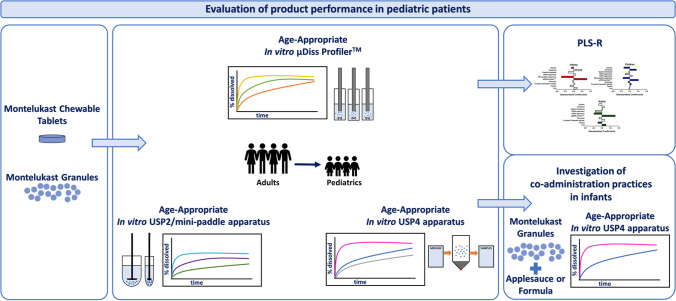

## INTRODUCTION

Dissolution tests have become an essential tool in drug development, from early to late stages, for a wide range of applications including quality control, formulation development, *in vitro-in vivo* relationships, establishment of clinically relevant specifications, and investigation of potential food effects (1, 2). To establish a good prediction of *in vivo* drug product performance, dissolution tests should be performed under physiologically relevant conditions by taking into consideration gastrointestinal (GI) fluid composition, gastric emptying, small intestinal transit times, and gastrointestinal volumes (2).

An abundance of adult biorelevant *in vitro* tools has emerged allowing a better prediction of *in vivo* drug product performance in adults (1–3). However, the applicability of adult biorelevant dissolution methods for evaluating drug product performance for the development of age-appropriate medicines remains questionable. The pediatric population undergoes continuous physiological and anatomical changes throughout childhood (4, 5), making it highly unlikely for a single pediatric dissolution test to be able to represent newborns, infants, and children (6). The development of age-appropriate biorelevant dissolution methods is needed, and its relevance has been highlighted by several research groups in recent publications (4, 5, 7–11).

A better understanding of the physiological and anatomical development changes is critical for further development and optimization of the current pediatric biopharmaceutics tools (4, 6). The composition of pediatric gastrointestinal fluids is affected by maturation changes, especially for newborns and young infants (5, 12, 13). Age-appropriate biorelevant media representative of newborns and infants (birth to 1 year) in the fasted and fed state has been proposed by Maharaj et al*.* (12). The proposed media gathers information on physiologically relevant components of GI fluids, such as pepsin concentrations, pH, osmolality, feed type for fed state media, concentrations of bile salts/lecithin and fat digestion products, and buffer capacity. Age-related changes in media composition have the potential to affect drug solubility and dissolution, and ultimately lead to altered drug performance (12, 14). This is especially important for poorly water-soluble compounds, for which drug solubility differences between pediatric and adult media are highly related to the physicochemical properties of the drug (logP, molecular weight, and ionization) and the composition of the biorelevant media (amount of bile salts/lecithin, and type of feeding) (14). The use of age-appropriate biorelevant media in dissolution experiments can improve the predictive power of the dissolution test, and an increased understanding of the *in vivo* drug behaviour, especially for formulations containing poorly water-soluble compounds.

The simulation of *in vivo* hydrodynamics equally requires careful consideration of *in vivo* gastrointestinal transit times, motility, and volumes*,* which are also affected by age (5, 15). The volume of fluids available in the GI tract will influence the disintegration and dissolution of a dosage form. For poorly water-soluble compounds, lower gastrointestinal fluid volumes might negatively affect the extent of drug dissolution (15). Both fasted and fed state volumes increase with age (15). In the fed state, gastrointestinal volumes are further affected by the type of food ingested, and the feeding schedules, which differ between the pediatric and adult population.

Gastric emptying times will determine the amount of time the drug will be in contact with the gastric fluids. For most drugs, the main site for absorption is the small intestine, which means that the gastric emptying rate will determine the rate and extent at which the drug will become available to be absorbed (16). Differences between pediatric subpopulations and adults are observed in terms of gastric emptying especially due to changes in food schedules, caloric content of meals, and differences in the types of food consumed (5, 17). GI tract motility patterns are a part of the *in vivo* hydrodynamics; *in vitro*, these patterns can be represented as flow rates/dip rates/agitation rates, according to the apparatus used (2).

Further complications arise in the pediatric population due to medicine manipulation practices, such as tablet crushing, capsule opening, and formulation mixing with different food and drinks that serve as vehicles for drug administration (18, 19). These drug handling practices can affect drug stability, solubility, and/or dissolution kinetics, which can result in differences in drug bioavailability between the adult and pediatric population, leading to possible issues related to drug efficacy and safety (10, 20, 21).

Age-appropriate biorelevant dissolution methods have been recently proposed to evaluate medicine co-administration with food and drinks (14). Drug dissolution was shown to be significantly affected by mixing the drug with different vehicles as well as by the time between preparation and testing of the vehicle-drug product mixtures (14).

Although some examples of age-appropriate biorelevant dissolution testing have started to emerge in the literature, there is still limited evidence regarding the most appropriate biopharmaceutics tools to be used in the pediatric population (4, 8, 11, 22). Reliable methods for the prediction of pediatric drug product performance are still needed. This will ensure that pediatric medicine development is supported by high-quality age-appropriate biopharmaceutics tools (4). Their applicability during the development of pediatric medicines is of extreme value to understand the biopharmaceutics risk of age-related changes in absorption and explore ‘what if’ clinical scenarios.

This study aims to fill this gap and develop age-appropriate *in vitro* biopharmaceutics tools to understand drug product performance in the pediatric population. Biorelevant dissolution set-ups were designed to understand how age and *in vivo* pediatric dosing scenarios impact *in vitro* drug dissolution performance.

Montelukast [a poorly water-soluble compound, clogP = 8.8 and pKa = 2.8 (basic) and pKa = 5.7 (acidic) (23, 24)] was chosen as a model drug due to the availability of two marketed pediatric formulations: Singulair^®^ chewable tablets (indicated for children) and Singulair^®^ granules (indicated for infants) (25, 26). Montelukast is indicated for the treatment of chronic asthma, and/or exercise-induced asthma, and allergic rhinitis. The pharmacokinetics (PK) of montelukast oral granules and chewable tablets have been studied in adult and pediatric patients (children and infants) (27–30). Singulair^®^ granules are recommended to be mixed with food vehicles (applesauce and formula) to facilitate administration (25, 26).

## MATERIALS AND METHODS

### Materials

Ultra-high temperature treated whole cow’s milk standardized to less than 4% fat was acquired from Sainsbury’s, UK. First infant milk formula (cow’s milk-based formula) (Cow & Gate, UK) and smooth applesauce were acquired from Sainsbury’s, UK.

Ammonium acetate, acetonitrile (ACN), and methanol [high-performance liquid chromatography (HPLC) grade], 37% hydrochloric acid, sodium hydroxide, sodium chloride, sodium acetate trihydrate, glacial acetic acid were purchased from Fisher Scientific (UK). Water was ultra-pure (Milli-Q) laboratory grade.

Montelukast sodium pharmaceutical secondary standard certified reference material, sodium oleate, pepsin (from porcine), and maleic acid were obtained from Sigma-Aldrich Company Ltd., UK. Singulair^®^ granules and chewable tablets (4 mg) (Merck Sharp & Dohme Ltd, UK) were obtained from UK and Belgium pharmacies. Sodium taurocholate (NaTC) (Prodotti Chimici Alimentari S.P.A., Italy), egg lecithin Lipoid EPCS (Lipoid GmbH, Germany), and glyceryl monooleate – Rylo Mg 19 (Danisco, Denmark) were obtained from the specified sources.

Regenerated cellulose [RC] membrane filters (13 mm, 0.45 µm) (Cronus^®^, UK), and glass microfiber syringe filters grade GF/D (13 mm, 2.7 µm) (Whatman™, UK), and porous full-flow polyethylene cannula filters (10 µm) (Quality Lab Accessories LCC, USA) were used. Polytetrafluoroethylene (PTFE) filter (13 mm, 0.45 μm) was purchased from Fisher Scientific (UK). Mackerey Nagel (MN) grade GF4 (MN GF-4) filters (15 mm, 1,4 μm) were purchased from VWR, UK. Glass microfiber filters grade GF/F (24 mm, 0.7 µm) and GF/D (24 mm, 2.7 µm) were from Whatman™, UK. Glass wool was obtained from Sigma-Aldrich Company Ltd., UK.

### Methods

#### Media Used for Dissolution Studies

Adult and pediatric biorelevant media were freshly prepared for each experiment, as described by Maharaj et al*.* (12). The formula used for the pediatric fed state gastric media representative of newborns (Pnc-FeSSGF) was prepared as per the manufacturer’s instructions: 1 scoop of powder (approximately 4.5 g) was added to 30 mL of boiled cooled water. For the two-stage dissolution testing, double concentrated FaSSIF-V2 was prepared with an additional amount of sodium hydroxide to achieve the final composition of FaSSIF-V2 (pH 6.5) after its addition to the gastric phase.

#### In Vitro Dissolution Studies

##### µDISS Profiler™ Dissolution Studies

**Fig. 1:**
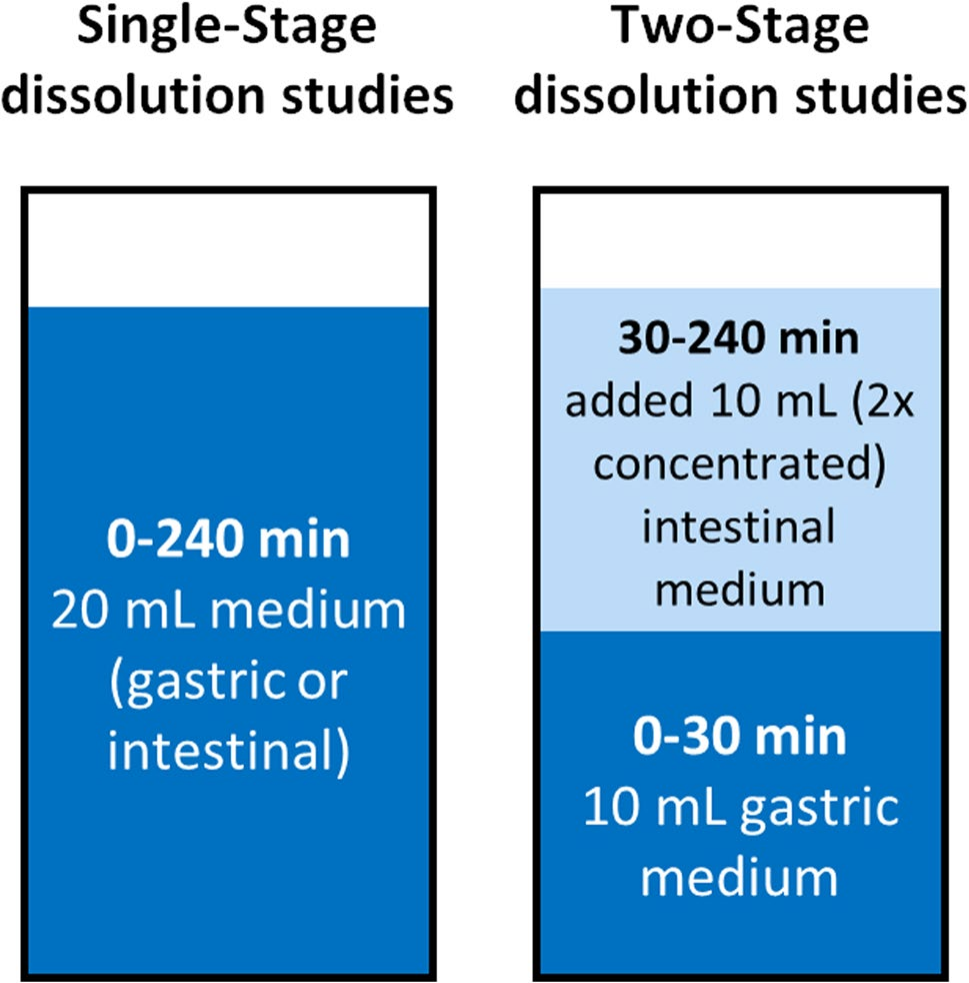
Schematic of the dissolution set-ups in the µDISS profiler™ dissolution studies

Dissolution studies were carried out using a μDISS Profiler™ (Pion Inc., MA). The 4 mg pediatric clinical dose of Singulair^®^ granules and crushed chewable tablet were down-scaled according to a final experimental volume of 20 mL, considering *in vitro* age-appropriate volumes (*i.e.* infants 150 mL, children 200 mL, and adults 500 mL). For infants, children, and adults, the down-scaled doses were 0.53, 0.4, and 0.16 mg, respectively. Dissolution studies were conducted either with a single-stage approach or a two-stage approach as presented in Fig. 1.

For the single-stage dissolution set-ups, the formulation was added into 20 mL of dissolution medium. For the two-stage dissolution, the formulation was initially added to 10 mL of the fasted gastric medium (*i.e.* fasted simulated gastric fluid (FaSSGF) for adults and children scenarios, and infant fasted gastric fluid (Pi-FaSSGF) in the infant scenario). At 30 min, 10 mL of double-concentrated fasted intestinal simulated fluid (FaSSIF-V2) was added to the dissolution vessel.

Samples were stirred at 300 rpm for all experiments and the concentrations of drug dissolved were monitored for 4 h. The temperature for all experiments was maintained at 37 °C by circulating water through a heating block mounted to the μDISS profiler™. Drug quantification was performed in situ by UV fiber optic dip probes (path length 5 mm, with wavelength 300 to 400 nm) connected to the Pion^®^ Rainbow Ultraviolet (UV) spectrometer system. Multiwavelength UV spectra were recorded at 30-s intervals. Calibration curves prepared in respective fresh media before the experiment (0.5 to 30 µg/mL), by appropriate dilutions of a 1 mg/mL stock solution of montelukast analytical standard in methanol. All experiments were performed in triplicate.

##### USP 2 Apparatus and Mini-Paddle Apparatus Dissolution Studies

Dissolution studies of 4 mg Singulair^®^ granules or crushed chewable tablets were performed with a single-stage approach in either a USP 2 apparatus (Agilent 708-DS Dissolution apparatus; Agilent, USA) or a mini-paddle apparatus where TruAlign 200 mL vessels and electropolished stainless-steel mini-paddles were used (Agilent, USA). Experiments were conducted at 37 °C. Volumes were selected according to the respective age group tested (Table I). The agitation rate in USP 2 apparatus was set at 50 revolutions per minute (rpm). For the mini-paddle apparatus, the agitation rate was set based on a speed factor of 2.5 between the paddle and mini-paddle hydrodynamics to reflect the agitation rate used in the USP 2 apparatus (31). Sample collection (2 mL samples withdrawn with corresponding medium replacement) took place at 5, 15, 30, 45, 60, 75, 90, 120, 180, and 240 min, using a glass syringe (Fortuna Optima^®^ fitted with a stainless tubing) through a cannula fitted with a 10 µm full-flow filter. After collection, aqueous-based samples were filtered through a 0.45-µm PTFE filter, treated (1000 µL of acetonitrile were added to 500 µL of the filtered sample), vortexed (HTZ, Cheshire, UK) for 1 min, and injected into the HPLC. Filter adsorption studies were performed in triplicate during preliminary studies. No adsorption issues onto the filters used in this study were observed. Milk- and formula-based samples were filtered through a 2.7-µm GF/D filter, treated with acetonitrile (1000 µL of acetonitrile was added to 500 µL of the filtered sample), vortexed for 1 min, and centrifuged (8000 rpm, 15 min, 4 °C) (Beckman Coulter J2-MC centrifuge, High Wycombe, UK). The supernatant was filtered through a 0.45-µm PTFE filter and injected into the HPLC. All experiments were performed in triplicate. To explore the dissolution performance of the drug products, and investigate if there were experimental issues (such as coning), dissolution tests were performed with Singulair^®^ granules in FeSSGF media (500 mL) with the USP 2 apparatus at three agitation rate conditions; 50, 75, and 100 rpm (data not shown).

**Table I.** Experimental Conditions Used in the Single-Stage Dissolution Experiments Conducted in the USP 2 and Mini-Paddle Apparatus

Age group	Apparatus	Gastric conditions			Intestinal conditions		
		Medium	Volume (mL)	Agitation (rpm)	Medium	Volume (mL)	Agitation (rpm)
Fasted state							
Infants	Mini-paddle	Pi-FaSSGF	75	125	FaSSIF-V2	150	125
Children	Mini-paddle	FaSSGF	100	125	FaSSIF-V2	200	125
Adults	USP 2	FaSSGF	250	50	FaSSIF-V2	500	50
Fed state							
Infants	Mini-paddle	Pnc-FeSSGF^a^/FeSSGF^b^	150	125	Pi-FeSSIF	200	125
Children	USP 2	FeSSGF	350	50	FeSSIF-V2	700	50
Adults	USP 2	FeSSGF	500	50	FeSSIF-V2	900	50

^a^Younger infants and ^b^Older infants

*FaSSGF*, adult fasted state simulated gastric fluids; *FaSSIF-V2*, adult fasted state simulating intestinal fluids; *FeSSGF*, adult fed state simulated gastric fluids; *FeSSIF-V2*, adult fed state simulated intestinal fluids; *Pi-FaSSGF*, pediatric fasted state gastric simulated fluids representative of infants (1–12 months); *Pnc-FeSSGF*, pediatric fed state simulated gastric fluids representative of newborns (0–28 days) fed cow’s milk-based formula; *Pi-FeSSIF*, pediatric fed state simulated intestinal fluids representative of infants

##### USP 4 Apparatus Dissolution Studies

**Dissolution Studies Simulating Fasted and Fed State**

Dissolution studies were conducted in an Erweka^®^ flow-through dissolution tester (USP 4 apparatus; model DFZ720, Erweka GmbH, Germany) connected to an Erweka^®^ Piston Pump (model HKP720). For the fasted state dissolution experiments, the USP 4 apparatus was equipped with Ø 22.6 mm cells (maintained at 37 ± 0.5 °C). A 5-mm size glass bead was positioned in the bottom of the cell, and glass beads (1 mm size) were added to the conical part of each cell. On the top of the cell, Whatman^®^ glass microfiber filters were used (GF/F and GF/D with pore sizes of 0.7 µm and 2.7 µm, respectively). During the experiment, 4 mg Singulair^®^ granules or 4 mg crushed chewable tablets were placed on top of the beads. Biorelevant media (adult and pediatric) were used as dissolution media. Duration of exposure to the various media and corresponding flow rates were appropriately selected to mimic the fasted and fed state transit times and gastrointestinal volumes of each subpopulation. Flow rates were selected to achieve a balance between the duration of the drug product exposure to the various media, and total fluid volumes, taking into account the lack of radial water flux *in vitro* (32). The *in vitro* conditions used are presented in Table II. Experiments were run in open-loop mode, with fresh media continuously passing through the cells (33). Samples were collected in volumetric cylinders and exchanged every 15 min, diluted, and assayed by HPLC. Experiments were run in triplicate.

**Table II.** Experimental Conditions Used in the Dissolution Experiments Conducted with the USP 4 Apparatus

Age group	Gastric conditions				Intestinal conditions			
	Residence time (min)	Flow rate (mL/min)	Volume (mL)	Medium	Residence time (min)	Flow rate (mL/min)	Volume (mL)	Medium
Fasted state								
Newborns (low)	0–45	2	90	Pn-FaSSGF	45–285	2	480	P50%-FaSSIF
Newborns (high)	0–45	2	90	Pn-FaSSGF	45–285	2	480	P150%-FaSSIF
Infants	0–30	4	120	Pi-FaSSGF	30–270	3	720	FaSSIF-V2
Children	0–30	8	240	FaSSGF	30–270	4	960	FaSSIF-V2
Adults	0–30	12	360	FaSSGF	30–270	4	960	FaSSIF-V2
Fed state								
Newborns	0–120	2	240	Pnc-FeSSGF	120–360	3	720	Pnc-FeSSIF
Younger infants	0–120	3	360	Pnc-FeSSGF	120–360	4	960	Pi-FeSSIF
Older infants	0–120	3	360	FeSSGF	120–360	4	960	Pi-FeSSIF
Children	0–165	3	495	FeSSGF	165–405	5	1200	FeSSIF-V2
Adults	0–90	8	720	FeSSGF	90–330	6	1440	FeSSIF-V2

**Dissolution set-ups: Newborns (low)**, newborns gastric Pn-FaSSGF followed by P50%-FaSSIF; **Newborns (high)**, newborns gastric Pn-FaSSGF followed by P50%-FaSSIF; **Media abbreviations:** *FaSSGF*, adult fasted state simulated gastric fluids; *FaSSIF-V2*, adult fasted state simulating intestinal fluids; *FeSSGF*, adult fed state simulated gastric fluids; *FeSSIF-V2*, adult fed state simulated intestinal fluids; *P150%-FaSSIF*, pediatric fasted state intestinal simulated fluids formulated with bile salt concentrations 150% (*i.e.* 4.5 mM) of adult levels; *P50%-FaSSIF*, pediatric fasted state simulated intestinal fluids formulated with bile salt concentrations 50% (*i.e.* 1.5 mM) of adult levels; *Pi-FaSSGF*, pediatric fasted state gastric simulated fluids representative of infants (1–12 months); *Pn-FaSSGF*, pediatric fasted state gastric simulated fluids of newborns (0–28 days); *Pnc-FeSSGF*, pediatric fed state simulated gastric fluids representative of newborns (0–28 days) fed cow’s milk-based formula; *Pnc-FeSSIF*, pediatric fed state simulated intestinal fluids representative of newborns (0–28 days) fed cow’s milk-based formula; *Pi-FeSSIF*, pediatric fed state simulated intestinal fluids representative of infants

For the fed state studies, USP 4 apparatus powder and granule cells were used (maintained at 37 ± 0.5 °C). A glass bead (5-mm diameter) was positioned in the apex of the flow-through cells. The lower conical part of the cell was filled with the formulation (4 mg Singulair^®^ granules or 4 mg crushed chewable tablets). Glass wool (0.10 g) was placed on top of the formulation on the lower part of the cell. On the top of each cell, 0.30 g of glass wool was added, followed by an MN GF-4 1.4 μm filter. Experimental conditions are described in Table II.

Aqueous-based samples were filtered through a 0.45-µm PTFE filter, treated (1000 µL of acetonitrile was added to 500 µL of the filtered sample), and injected into the HPLC. Milk- and formula-based samples were treated (1000 µL of acetonitrile was added to 500 µL of the filtered sample), vortexed for 1 min, and centrifuged (8000 rpm, 15 min, 4 °C) and the supernatant was filtered through a 0.45-µm PTFE filter and injected into the HPLC.

**Dissolution Studies Simulating Medicine Co-administration Practices in Infants**

According to the British National Formulary for Children (BNF-C), Singulair^®^ granules may be swallowed or mixed with cold, soft foods (not liquid), and taken immediately (25). In the present study, in addition to the fasted direct administration of formulation scenario (as described in the previous subsection), two additional scenarios were investigated: mixing formulation with formula milk or applesauce. These vehicles were selected due to their use of dosing vehicles for the administration of montelukast granules in the *in vivo* PK studies in infants (30–32). Because the prandial state of the patients was not reported in the *in vivo* study, both fasted and fed intestinal conditions were simulated *in vitro*. The USP 4 apparatus conditions for the simulation of medicine co-administration practices in infants are presented in Table III. In the scenarios where the drug was mixed with vehicles, each sample was prepared by the addition of the formulation to 5 mL of applesauce/formula measured with a plastic syringe, followed by mixing with a stainless-steel spatula. On the top of the USP 4 apparatus cell (Ø 22.6 mm cells, maintained at 37 ± 0.5 °C), Whatman^®^ glass microfiber filters, GF/F (0.7 µm) and a GF/D (2.7 µm), separated by a layer of 0.10 g of glass wool were used for sample filtration. The remaining dissolution conditions (temperature, sampling points, sample collection, and treatment) were performed as described in the previous subsection.

**Table III.** *In Vitro* Conditions Used in the USP 4 Apparatus to Study Drug and Food Co-administration Practices

Scenario	Vehicle	Gastric conditions				Intestinal conditions			
		Residence time (min)	Flow rate (mL/min)	Volume (mL)	Medium	Residence time (min)	Flow rate (mL/min)	Volume (mL)	Medium
FaG/FaI	Direct (no vehicle)	0–30	4	120	Pi-FaSSGF	30–270	3	720	FaSSIF-V2
FaG/FaI	Applesauce						3	720	FaSSIF-V2
FaG/FeI							4	960	Pi-FeSSIF
FaG/FaI	Formula						3	720	FaSSIF-V2
FaG/FeI							4	960	Pi-FeSSIF

*FaG/FaI*, fasted gastric conditions followed by fasted intestinal conditions; *FaG/FeI*, fasted gastric conditions followed by fed intestinal conditions

#### Chromatographic Conditions for Drug Quantification

HPLC analysis was performed using a Hewlett Packard Series 1100 system equipped with an autosampler, temperature-regulated column compartment, quaternary pump, and diode array detector (DAD) (Agilent Technologies, UK). The chromatographic method used for the quantification of montelukast was a modification of the method by Raju NK *et al*. (34). A C18 column was used (Kromasil 100 Å C18 4.6 × 250 mm, 5 µm). The injection volume was 100 µL. The temperature of the column compartment and the sample tray was set at 20 °C. The mobile phase consisted of methanol: ammonium acetate buffer (50 mM; pH = 5.5) (90:10 v/v); the flow rate was 1 mL/min and the DAD detector was set at *λ* = 284 nm. The run time was set at 10 min. Quantification of montelukast in samples was performed with calibration curves of freshly prepared standard solutions (calibration curve range: 0.3–40 μg/mL). Standards were prepared in the medium of interest for each experiment by appropriate dilution of a 1 mg/mL stock solution of montelukast analytical standard in methanol. The limit of detection and the limit of quantification were 0.12 and 0.3 μg/mL, respectively.

#### Statistical Analysis of Dissolution Data

To describe and compare the obtained drug dissolution profiles, the linear trapezoidal method was used to calculate the area under the curve for each dissolution profile over 4 h (AUC_0-4 h_) using Microsoft Excel 2016, Office 365^®^ (Microsoft, USA). This allowed the use of a single representative value of drug dissolution to compare the different scenarios tested.

##### Multivariate Statistical Analysis of *In Vitro* Dissolution Studies

Dissolution studies investigated in the µDISS profiler™, USP 2 apparatus, mini-paddle apparatus, and USP 4 apparatus (fasted and fed state with direct administration of the formulation) were analyzed with partial least squares regression (PLS-R) analysis. The PLS-R was used to correlate the AUC_0-4 h_ (%diss⋅h) (dependent variable) with the explanatory variables that described the dissolution set-ups used in the experiments. PLS-R analysis was performed with XLSTAT Software (an add-in for Excel, Microsoft^®^). The explanatory variables were (i) gastrointestinal compartment (gastric, intestinal, gastric/intestinal); (ii) prandial state (fasted or fed); (iii) formulation (granules or chewable tablets); and (iv) hydrodynamics (µDISS profiler™, USP 2 apparatus, or mini-paddle and USP 4 apparatus). All explanatory variables were set as categorical variables. PLS-R analysis was grouped based on age: infants, children, and adults. For newborns, since the montelukast drug label does not recommend the use of this formulation in this age group, only exploratory dissolution studies were performed in the USP 4 apparatus; therefore, this age group was not included in the PLS-R analyses (26). The quality of the model was evaluated by the square of the coefficient of determination (*R*^2^) and goodness of prediction (*Q*^2^) (34–36). Values close to 1 are indicative of good fit and prediction power, respectively. Full cross-validation (leave-one-out procedure) was used to develop and evaluate the regression model. The optimum number of calibration factors for each model was selected based on the optimum predictability of the model and predicted residual error sum of squares (PRESS) (35–37). Standardized coefficients, that indicate the relative effect (positive or negative) of their corresponding variables on the response, were generated for each independent variable (35–37. The variable importance in projection (VIP) value was used to evaluate the importance of each factor on the model (35–37). Model variables with VIP values > 1 were evaluated as the most important in explaining the variation in the dependent variable, while values between 0.7 and 1 were considered to have a moderate impact. Values < 0.7 were deemed as not significant for the prediction of the dependent variable (35–37).

##### Impact of Medicine Co-administration Practices on Drug Dissolution Performance in Infants

Dissolution data from the studies simulating medicine co-administration practices in infants were analyzed. One-way analysis of variance (ANOVA) with a post hoc Tukey’s multiple comparisons test was performed (GraphPad Prism^®^ v.6 Software) to investigate statistically significant differences (*p* < 0.05 noting significance level) in the *in vitro* AUC_0-4 h_ obtained for the different dosing scenarios tested. Plasma concentration–time of montelukast granules (4 mg) co-administered with formula or applesauce to 3 infant cohorts (formula: 1 to 3 months; applesauce: 3 to 6 months and 6 to 24 months) were obtained from the literature (27–30). The observed PK profiles found in the literature were digitalized with the WebPlotDigitizer^®^ v4.1 software. Non-compartmental PK data analysis of the mean plasma concentration–time profile for each age group was performed with PKSolver^®^ (add-in program for Microsoft Excel^®^) (39). *In vitro* AUC_0-4 h_ was compared to *in vivo* AUC_0-24 h._

## RESULTS

### In Vitro Dissolution Studies

#### µDISS Profiler™ Dissolution Studies

**Fig. 2:**
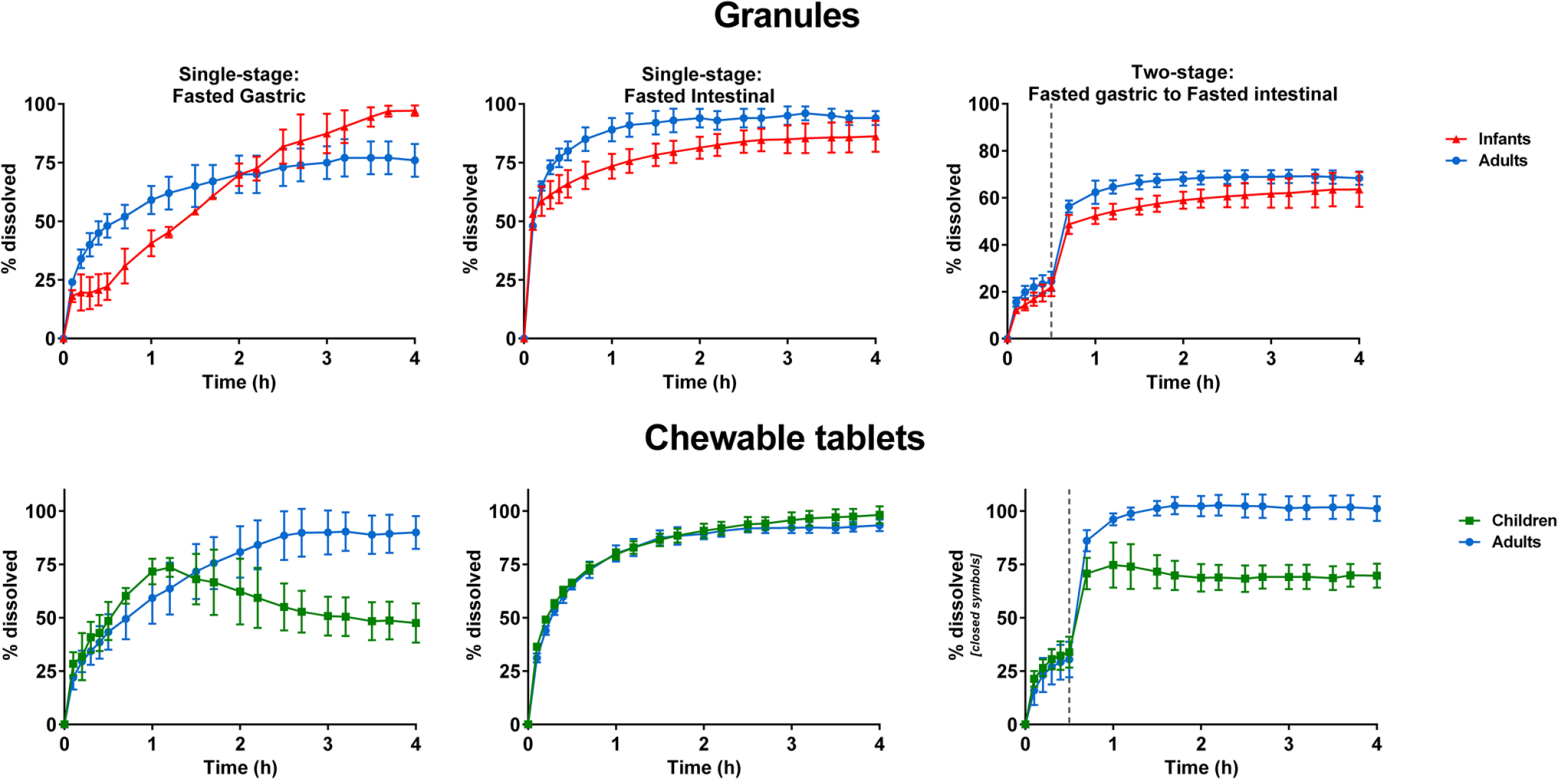
Mean montelukast % dissolved [± standard deviation (SD)] from Singulair^®^ granules and crushed chewable tablets in infants, children, and adults conditions with the µDISS Profiler™

Dissolution profiles of montelukast in the μDISS Profiler™ are presented in Fig. 2. The results show that montelukast dissolution was affected by dose/volume ratio and media changes that were used to represent age-related changes.

In all the fasted gastric state set-ups, when testing with Singulair^®^ granules, drug dissolution rate was initially (for the first 2 h) faster in adults when compared to infants. However, at the end of 4 h of dissolution, a higher % drug was dissolved in the infants’ set-up (97% drug dissolved) compared to adults’ set-up (76% drug dissolved). This observation was likely related to a higher dose to volume ratio representing infants’ dosing and gastrointestinal conditions after the administration of montelukast. Additionally, the infant fasted gastric simulated medium (Pi-FaSSGF) contains a lower concentration of bile salts and lecithin in comparison to the adults fasted gastric simulated medium (FaSSGF), which has been shown to negatively affect montelukast solubilization (14).

In the FaSSGF children set-up, a slight decrease in the concentration was observed after 1.30 h of dissolution of montelukast from the chewable tablets formulation (maximum concentration reached was approximately 15 µg/mL at 1.20 h, which then decreased to 9 µg/mL at the end of the dissolution test). This could indicate a small extent of precipitation due to the high dose/volume ratio used to represent children’s dosing and gastrointestinal conditions, which is higher than the solubility of montelukast in FaSSGF (approximately 0.8 µg/mL) (14). As it is unlikely that montelukast would remain in the stomach for more than 1.30 h, this observation was not further investigated. Although a higher dose/volume ratio was present in the infants’ set-up (dissolution testing with granules) when compared to the children set-up (dissolution testing with crushed chewable tablets), no sign of precipitation was observed for infants. These results reveal that montelukast dissolution in the fasted gastric state was affected by the type of formulation, which can also be observed in the adult set-ups (granules in comparison to crushed chewable tablets).

In the simulated intestinal fasted state, drug dissolution from both formulations was fast and complete between all age groups (> 85% dissolved at 4 h).

In the two-stage set-up (simulation of fasted gastric to intestinal dissolution), a smaller % drug dissolved from Singulair^®^ granules (approximately 70% drug dissolved) was observed in comparison to single-stage dissolution in FaSSIF-V2 (approximately 90–100% drug dissolved), in both adults and children conditions. It is likely that media change, and montelukast lower solubility in simulated gastric fluids than in intestinal fluids, contributes to this difference (14). For the Singulair^®^ crushed chewable tablets, % drug dissolved at 4 h was approximately 100% in the adult fasted two-stage and single-stage intestinal dissolution set-ups. In children’s two-stage dissolution set-up with Singulair^®^ crushed chewable tablets, the % drug dissolved was lower than when testing under adult biorelevant conditions. The different dissolution behaviour in the two-stage fasted gastric set-up indicates that formulation characteristics might also impact montelukast’s dissolution.

#### USP 2 Apparatus and Mini-Paddle Apparatus Dissolution Studies

**Fig. 3:**
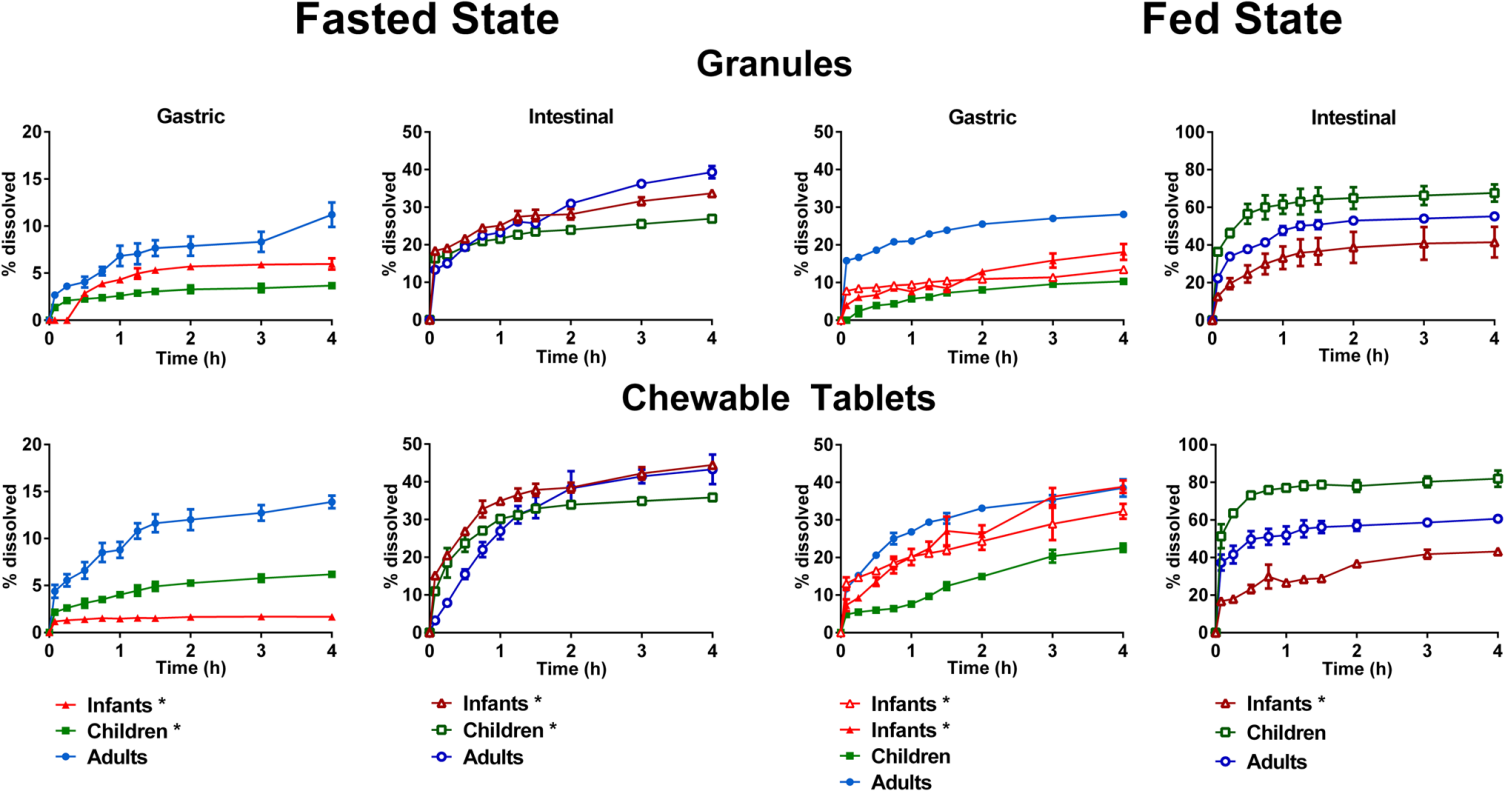
Mean montelukast % dissolved (± SD) from Singulair^®^ 4 mg granules and chewable tablets in adults, children, and infants’ conditions using the USP 2 apparatus and mini-paddle apparatus (set-ups marked with * denote the use of the mini-paddle apparatus)

Montelukast dissolution from the Singulair^®^ granules and crushed chewable tablets under adult and pediatric biorelevant conditions simulated in the USP 2 apparatus and mini-paddle apparatus is presented in Fig. 3.

In all the set-ups tested, montelukast dissolution from both test formulations was not complete (Fig. 3). In the fasted gastric simulated state, montelukast dissolution from both formulations was limited by its solubility (approximately 0.8 µg/mL in adult fasted gastric simulated fluids (FaSSGF)(14). Montelukast dissolution extent was low for all age groups, ranging approximately 2 to 15% dissolved at 4 h.

In the fed gastric simulated state, % drug dissolved was influenced by the volume of media used. An overall higher dissolution was observed in the adult set-up (28 and 38% drug dissolved from Singulair^®^ granules and crushed chewable tablets, respectively), in comparison to the children and infants’ dissolution set-ups.

In the simulated fasted intestinal state, the lowest % of drug dissolved at 4 h was observed in the children’s scenario, for both formulations. The % drug dissolved from the Singulair^®^ granules was always lower when compared to the dissolution of Singulair^®^ crushed chewable tablets.

In the simulated fed intestinal set-ups (adults and pediatrics), for both formulations, the highest extent of drug dissolved at 4 h was observed in the children set-up, and the lowest in the infants’ set-up.

Montelukast dissolution from both formulations was not complete under the scenarios tested and its dissolution was not limited by its solubility. Dissolution studies with montelukast granules were performed at different agitation rates (50, 75, and 100 rpm) in 500 mL of fed gastric simulating fluids (FeSSGF), and revealed that agitation rate does not have an impact on the % montelukast dissolved confirming that the low dissolution observed is not attributed to coning (data not shown).

#### USP 4 Apparatus Dissolution Studies: Simulating the Fasted and Fed State

**Fig. 4:**
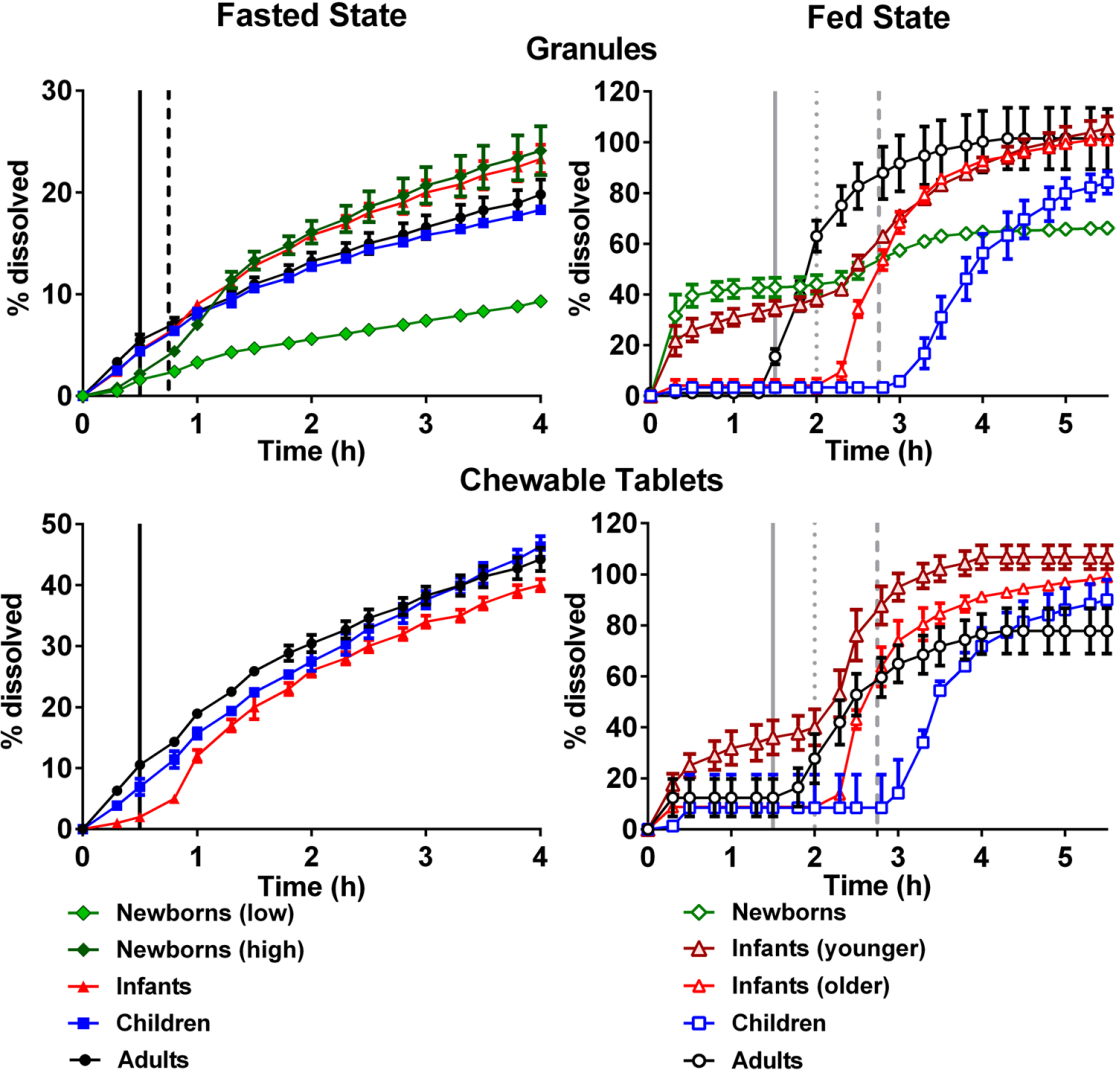
Mean montelukast % dissolved (± SD) from 4 mg Singulair^®^ granules and 4 mg crushed chewable tablets using the USP 4 apparatus (with open-loop mode). The vertical lines represent the time for media change for the fasted state (black line—adults; dashed black lines—newborns) and fed state (grey line—adults; dotted grey lines—infants and newborns; dashed grey lines—children)

Drug dissolution from montelukast granules and crushed chewable tablets in all the fasted state simulated set-ups was slow and incomplete (Fig. 4), and showed a slower dissolution rate during the gastric phase compared to the intestinal phase.

The highest % of montelukast dissolved from Singulair^®^ granules was obtained under the newborns’ (high) simulated conditions (Pn-FaSSGF to P150%-FaSSIF). On the other hand, the lowest % of drug dissolved was obtained for newborns (low) set-up (Pn-FaSSGF to P50%-FaSSIF). Since the differences between newborns’ dissolution set-ups are related to the media composition, the dissolution results appear to be sensitive to the content of bile salts and lecithin in the biorelevant media used. This is in accordance with drug solubility studies previously conducted, in which it was shown that lecithin and bile salts concentration have a positive effect on drug solubilization (14).

In the fasted state, the dissolution extent of montelukast crushed chewable tablets was always higher when compared to the dissolution extent from the Singulair^®^ granules. When testing the granules formulation, drug dissolution was slightly higher for the infants’ scenario than the adults’ one. On the contrary, for the Singulair^®^ crushed chewable tablets, the % drug dissolved was the lowest in the infants’ dissolution scenario. This might be related to the different effect of the continuous media flow in the two formulations inside the USP 4 apparatus cell (that would depend on the weight of the granules *vs* weight of the chewable tablets formulation).

In the simulated fed state, the % drug dissolved from both formulations was associated with a higher variability than when testing under simulated fasted state conditions. Dissolution in the gastric fed simulated state appeared to be hindered by the media viscosity, which was observed as a result of the formulation behaviour in the presence of fed gastric medium which contains 50% milk formula or cow’s milk. The increase in viscosity was evident from the fact that when testing the fed state dissolution set-ups with the tablet cell used in the simulated fasted state set-ups clogging of the cells and leakage of medium was observed. Therefore, the dissolution studies in the simulated fed state were conducted in the USP 4 apparatus granule and powder cell with 2 separate layers of glass wool. Montelukast dissolution was negligible in the simulated fed gastric phase (for both formulations tested) (FeSSGF, pH 5) (*i.e*. older infants, children, and adult’s dissolution set-ups). The effect of the medium pH was also evident when dissolution in adult FeSSGF (pH 5) was compared to dissolution in the pediatric fed state medium containing formula (Pnc-FeSSGF, pH 5.7, *i.e.* newborns’ and young infants’ dissolution set-ups). The % drug dissolved increased significantly in the simulated fed gastric pediatric conditions which is related to montelukast’s ionization properties (amphoteric; pKa (basic) 2.8 and pKa (acidic) 5.7) and its higher solubility in Pnc-FeSSGF than in FeSSGF (3.5 mg/mL and 2.4 mg/mL, respectively) (14). The introduction of fed state simulated intestinal media notably increased the dissolution rate of montelukast from both formulations in all set-ups tested. For all the simulated fed state dissolution tests performed with the exception of the tests performed in the newborns’ set-up, more than 70% of the drug was dissolved within 2 h of the introduction of the fed state simulated intestinal medium. Contrary to the simulated fasted state dissolution studies, a clear trend between the dissolution of granules and crushed chewable tablets was not observed, and similar results were achieved for all conditions. Since dissolution was influenced by the introduction of the fed intestinal simulating fluids, the longer gastric residence times in the infants and children scenarios (compared to the adult ones) resulted in a delay of drug dissolution.

#### Multivariate Statistical Analysis of *In Vitro* Dissolution Studies

**Fig. 5:**
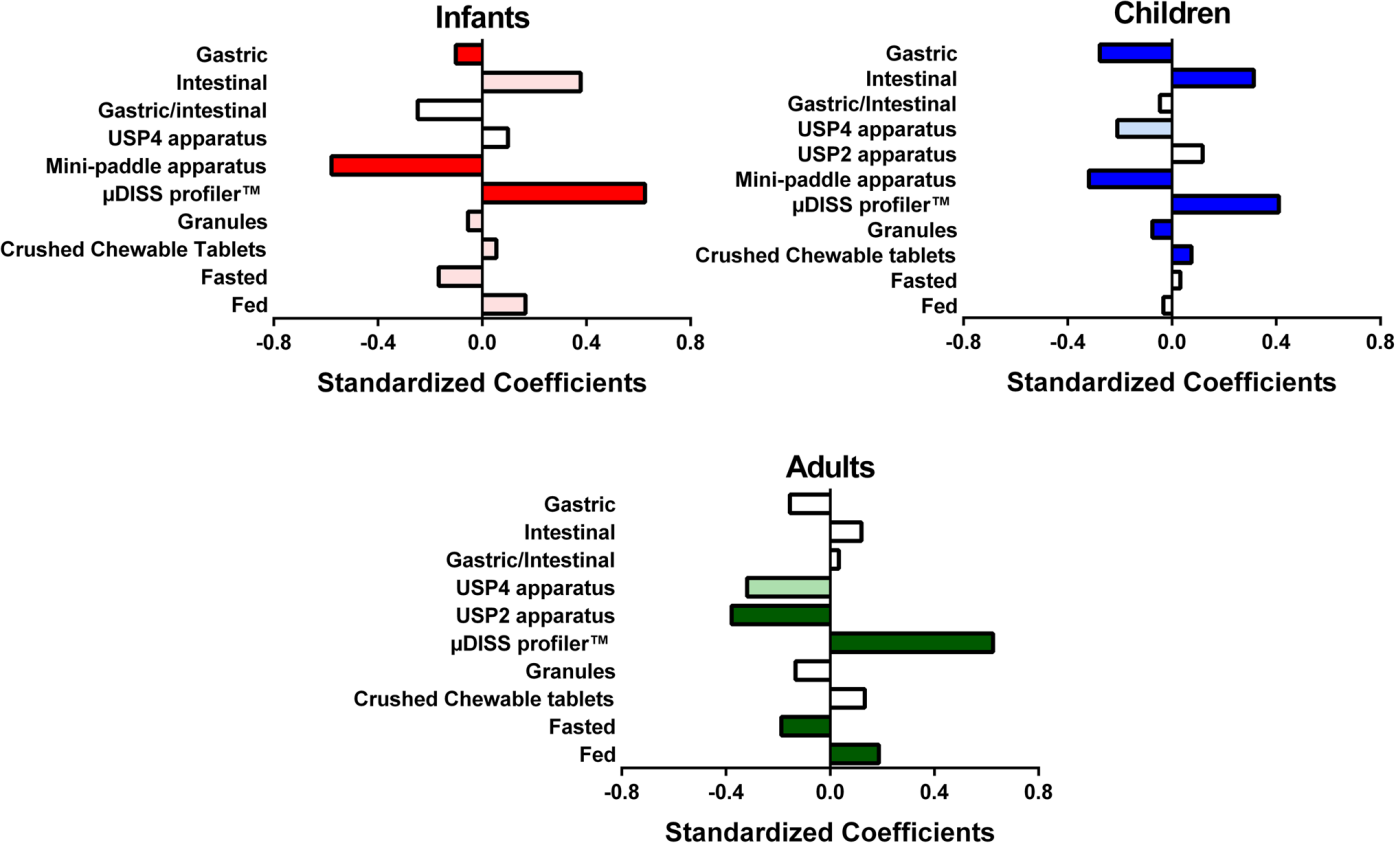
Standardized coefficients corresponding to the variables studied for the dissolution of montelukast. Colour denotes coefficients with a moderate (lighter colour, VIP between 0.7 and 1) and significant (darker colour, VIP > 1) impact on the response

Results from the PLS-R analysis, conducted to understand the effect of *in vitro* hydrodynamics on the dissolution of montelukast from the Singulair^®^ granules and crushed chewable tablets in each age group (infants, children, and adults), are presented in Fig. 5.

The PLS-R model for the infant’s dissolution set-ups was defined by 2 components and showed a good fit to the experimental values (*R*^2^ = 0.82) and good predictive power (*Q*^2^ = 0.74). Montelukast dissolution in infants’ set-ups was significantly affected by the dissolution apparatus used to perform the dissolution tests (µDISS profiler™ showed a positive effect, and mini-paddle apparatus showed a negative effect). The statistical analysis also revealed that the simulation of the gastrointestinal compartment is an important variable affecting the dissolution of montelukast. Overall, simulation of the gastric compartment showed a significant negative impact on drug dissolution, and simulation of the intestinal compartment showed a moderate positive impact on drug dissolution (Fig. 5). Moderate impact (VIP between 0.7 and 1) on the dissolution of montelukast was also observed from the simulation of the prandial state (fasted state: negative impact; fed state: positive impact), and the formulation type (granules: negative impact; crushed chewable tablets: positive impact).

The PLS-R model for the children’s dissolution set-ups was defined by 3 components and showed a good fit to the experimental values (*R*^2^ = 0.79) and a good predictive power (*Q*^2^ = 0.87). The statistical analysis revealed that simulation of the gastrointestinal conditions (gastric compartment: negative impact, intestinal compartment: positive impact), *in vitro* hydrodynamics (µDISS Profiler™: positive impact), and formulation type (granules: negative impact; crushed chewable tablets: positive impact) were the factors with the most significant impact on drug dissolution in this age group (Fig. 5). The hydrodynamics of the USP 4 apparatus showed a moderate negative impact (VIP between 0.7 and 1) on the montelukast dissolution for the children scenarios testing.

The PLS-R model for adult’s dissolution set-ups was defined by 3 components and showed a good fit to the experimental values (*R*^2^ = 0.86) and good predictive power (*Q*^2^ = 0.78). The statistical analysis revealed that simulation of the prandial state (fasted state: negative impact; fed state: positive impact), and *in vitro* hydrodynamics (µDISS Profiler™: positive impact; USP 2 apparatus: negative impact) were the factors with the most significant impact on drug dissolution (Fig. 5). As observed for children, the hydrodynamics in the USP 4 apparatus showed a moderate negative impact on the dissolution of montelukast.

### Impact of Medicine Co-administration Practices on Drug Dissolution Performance in Infants

**Fig. 6:**
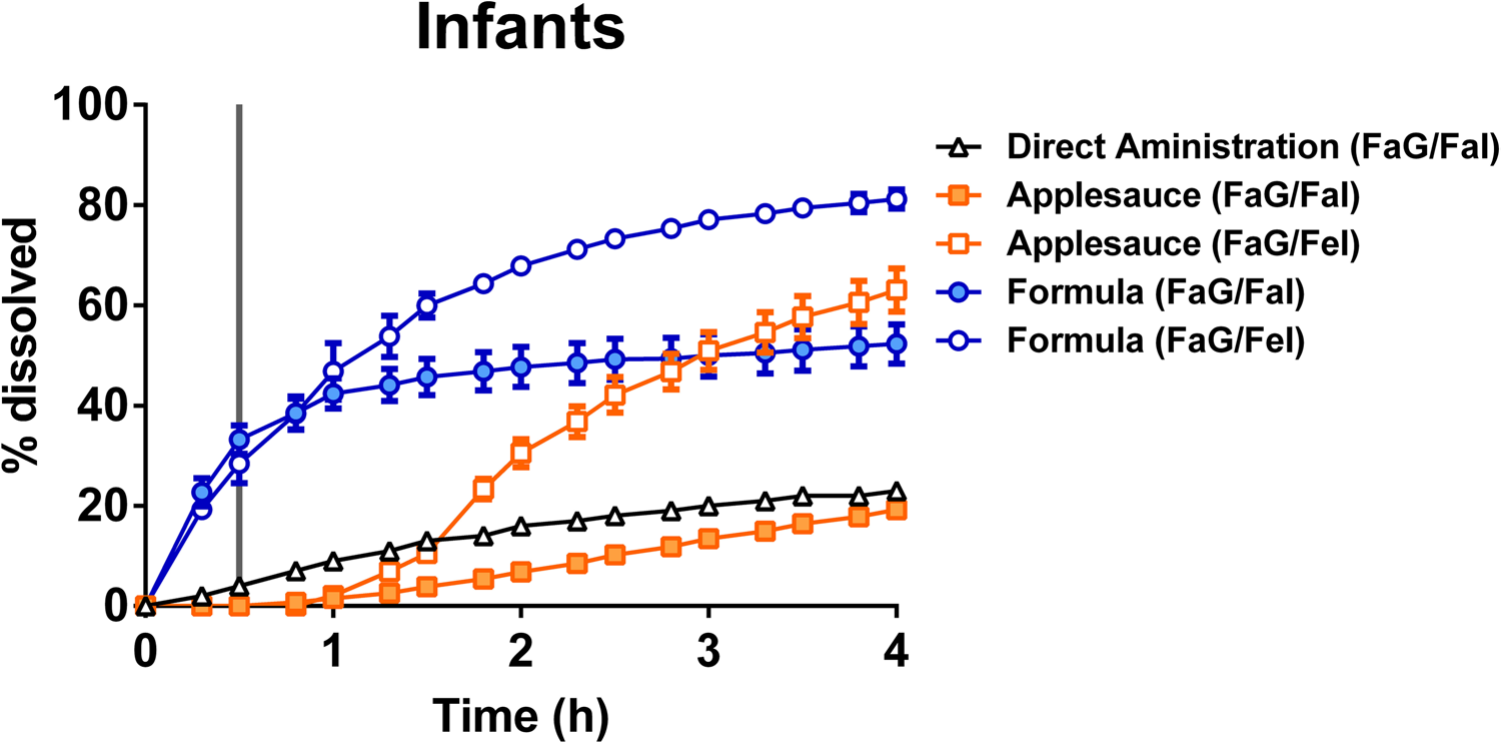
Mean montelukast % dissolved (± SD) from Singulair^®^ 4 mg granules in infants set-up tested with the USP 4 apparatus (open-loop mode). The straight vertical line represents the time for media change

**Fig. 7:**
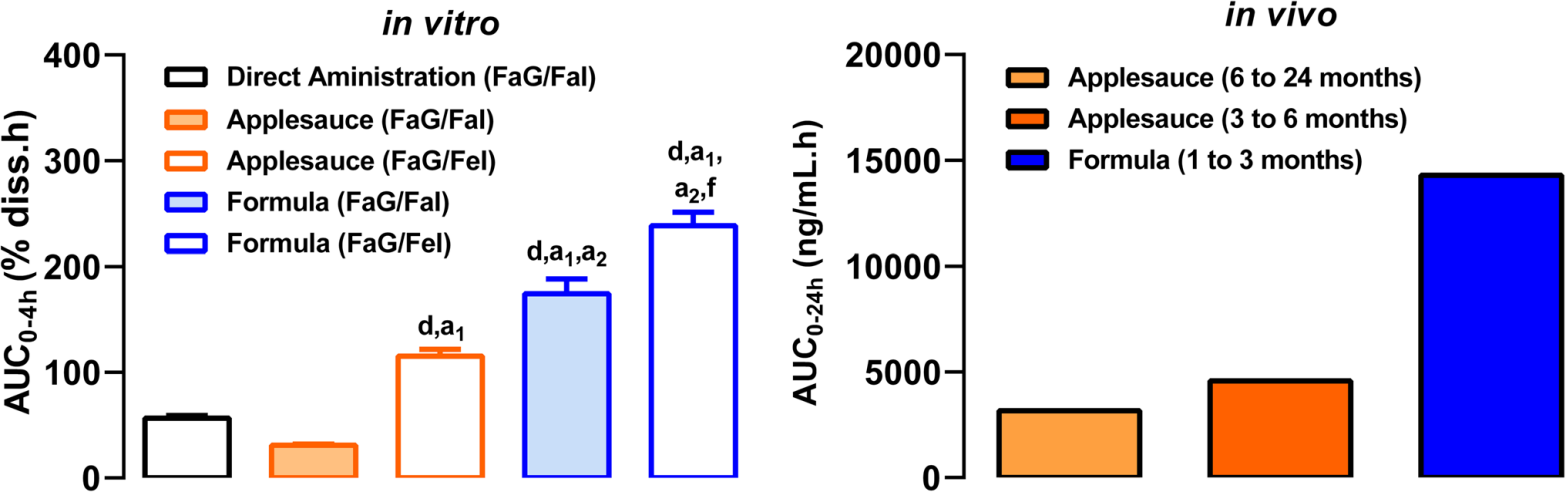
*In vitro* AUC_0-4 h_ (± SD) of Singulair^®^ oral granules calculated from montelukast dissolution profiles. Symbols denote a statistical difference in AUC_0-4 h_ between (d) FaG/FaI; (a_1_) applesauce (FaG/FaI); (a_2_) applesauce (FaG/FeI); (f) formula (FaG/FaI); *in vivo* mean AUC_0-24 h_ after administration of Singulair^®^ granules (4 mg) to 1 to 3 months infants with formula; 3 to 6 months and 6 to 24 months infants with applesauce (30–32, 37)

The effect of medicine co-administration practices on drug dissolution in infants’ set-ups is presented in Fig. 6. The *in vitro* AUC_0-4 h_ of the dissolution profiles and the *in vivo* AUC_0-24 h_ are presented in Fig. 7. The dissolution profile of the *in vitro* simulation of montelukast granules co-administration with applesauce showed a delay in the dissolution of montelukast during the first hour of the dissolution test, when compared with the scenario of the direct administration of the formulation (Fig. 6). This effect has also been observed in a previous study by Martir et al. and it is hypothesized to be related to the presence of starch in applesauce (11). The applesauce forms a gel around the formulation, which due to an increase in viscosity negatively affects the dissolution of montelukast granules (11).

The *in vitro* AUC_0-4 h_ of the dissolution profiles of the direct administration of the formulation scenario was significantly lower when compared to the co-administration with applesauce (fasted gastric/fed intestinal conditions) and formula (fasted gastric to fasted/fed intestinal conditions) (Fig. 7).

Dissolution from fasted gastric to fasted and fed intestinal state (represented by FaG/FaI and FaG/FeI, respectively) were simulated to investigate whether medicine co-administration with a vehicle could induce a food effect in infants (40). Simulating intestinal fed conditions after formulation co-administration with a food vehicle is especially relevant for younger populations, such as infants, due to the possibility of administration of the drug with small amounts of food which could potentially trigger the fed state. The results show that the *in vitro* AUC_0-4 h_ of the dissolution profile was significantly higher for the testing under fed state simulating intestinal conditions in comparison to the fasted state simulating intestinal conditions (Fig. 7). The *in vitro* studies reflected the *in vivo* observations, where higher drug exposure was found after mixing the formulation with formula instead of applesauce (Fig. 7).

Overall, it was observed that co-administration of Singulair^®^ granules with food and drink vehicles significantly affects the dissolution of montelukast. The choice of simulated intestinal conditions was shown to affect drug dissolution behaviour, with a higher % drug dissolved at 4 h when testing under fasted gastric to fed intestinal conditions (*i.e.* FaG/FeI).

## DISCUSSION

In this study, we observed that montelukast dissolution was strongly influenced by age-related changes of *in vitro* hydrodynamics. The *in vitro* hydrodynamics (related to each dissolution apparatus tested) was one of the most significant parameters impacting drug dissolution, which is related to the simulation of gastrointestinal hydrodynamics, such as gastrointestinal volumes and agitation properties. To achieve a predictive dissolution method for testing pediatric medicines, exploration of dissolution conditions (*e.g.* appropriate dissolution medium, and hydrodynamics), as performed in this study, is essential. The mimicking of *in vivo* age-related dissolution conditions is complex to reproduce in an *in vitro* system as the *in vivo* dissolution in the GI tract is a dynamic process (41). Mechanisms that influence *in vivo* dissolution include physical shear, grinding forces, and pressure exerted by peristaltic movements in the GI tract. In this study, the dissolution characterized by the apparatus used is only able to capture a fraction of these complex mechanisms offered by the GI tract (41). Optimization of *in vitro* hydrodynamics for the pediatric population with regard to simulation of *in vivo* GI tract hydrodynamics should be further optimized as *in vivo* data in the pediatric population becomes available.

In all set-ups, drug dissolution was positively impacted when tested in the fasted state in the µDISS profiler™. Montelukast dissolution in infants was negatively affected by the hydrodynamics in the mini-paddle apparatus. For the pediatric dissolution set-ups, the second most significant variable studied was the simulation of single-stage gastric (negative impact) and intestinal compartments (positive impact), which is in accordance with montelukast’s physicochemical properties (amphoteric; clogP 8.8; pKa (basic) 2.8 and pKa (acidic) 5.7), and its higher solubility in simulated intestinal fluids (FaSSIF-V2) compared to simulated gastric fluids (FaSSGF) (23, 24). In the adult’s set-ups, the simulation of the prandial state was also an important factor, with the simulation of fed state conditions showing a positive impact on the dissolution of montelukast.

Montelukast dissolution from both formulations in the USP 2/mini-paddle apparatus was not complete in the different scenarios tested, whereas a faster dissolution was observed in the studies with the µDISS profiler™. For example, when comparing the fasted gastric state dissolution profiles of granules in the infant’s scenario, a fast dissolution in the first 20 min is observed with the µDISS profiler™ (Fig. 2), whereas dissolution is negligible with the mini-paddle set-up (Fig. 3). The low extent of dissolution observed in the USP 2/mini-paddle apparatus could be potentially related to aggregation issues related to the montelukast’s very high lipophilicity (clogP of 8.8), the wettability of montelukast tested formulations, and/or other formulation characteristics (*e.g.* surface area and particle size distribution) (23, 42). Before dissolution testing, filter adsorption studies were performed, and no adsorption into the filters used was observed. No other measures were taken during dissolution studies, as previous studies have not reported this issue when working with montelukast and its formulations *in vitro* (9, 23). Overall, dissolution of montelukast appeared to be affected by formulation aspects and age-related media composition and volumes. Montelukast showed an overall slow and incomplete dissolution from both test formulations, especially when tested under fed gastric conditions. Solubility was not a limiting factor in this set-up, and coning was not observed (visually). For formulations exhibiting poor wettability, it may be necessary to increase the level of surfactants in the dissolution medium to obtain reproducible dissolution results (43). This option was discarded in our studies, due to loss of biorelevant simulating conditions. The differences in formulation dissolution with the apparatus used in this study are reflected in the statistical (PLS) analysis, which revealed that the *in vitro* hydrodynamics (*in vitro* apparatus) is an important factor influencing the dissolution of montelukast *in vitro*. In order to understand the most biopredictive set-ups, data could be further incorporated into Physiologically Based Pharmacokinetic (PBPK) models (5).

Hydrodynamic conditions differ between dissolution apparatus. The *in vitro* hydrodynamics in the different apparatus used in this study are not only related to the agitation rate, type of agitation rate (rpm *vs* flow rates), but also to the vessel size and shape, volume available for dissolution, shape of the agitator/paddle/beads, *etc*. All methods can be explored in a step-wise/cascade approach during drug development in order to achieve biorelevance and clinical relevance. Exploration can be performed from simpler methods (*i.e.* USP apparatus, µDISS profiler™, *etc*.) to more complex methods (such as the TNO TIM-1 and TinyTIM models, which are dynamic multicompartment systems simulating the human GI tract) (41). Aspects to consider during dissolution method development depend on formulation factors and complexity (*e.g.* immediate-release (IR) dosage forms *vs* modified-release (MR) dosage forms) and on drug factors (*e.g.* highly soluble compounds *vs* poorly soluble compounds). For example, the USP 4 apparatus has been used for the characterization of MR formulations performance (as sequential media changes and pH shifts can be easily applied), and for the characterization of formulations of poorly soluble compounds (as sink conditions can be achieved when used in open-loop mode) (41).

The obtained *in vitro* AUC results for the investigation of the effect of medicine co-administration practices are in line with the *in vivo* AUC observations (Fig. 7), with a higher drug exposure being observed when the formulation was mixed with formula than when mixing with applesauce. The pediatric clinical PK data was from pediatric asthmatic patients. To the best of our knowledge, there is no evidence that asthma affects the GI physiology and ultimately drug absorption; therefore, it was assumed that the patient PK data is representative of healthy pediatric patients in regard to drug absorption. In the montelukast PK studies, the infants’ prandial state was not recorded, on the basis that montelukast does not show a food effect in adults (30–32, 44). However, the mechanisms behind food effects in the pediatric population are still unknown (20). The definition of fasted and fed state in infants is complex since the high frequency of meals might lead to the continuous presence of food remnants in the stomach and/or intestine (13). Little information is available on the amount of food that is necessary to trigger a food effect in newborns and young infants. Therefore, a food effect should not be disregarded in infants, even in cases where an adult food effect is not observed (20). Considering this, dissolution studies were performed under simulated fasted gastric to fasted intestinal conditions and simulated fasted gastric to fed intestinal conditions, and a higher % drug dissolved was observed in the fasted gastric to fed intestinal conditions testing, which is related to a higher drug solubility in fed state simulated media (14). The dissolution results are in accordance with a recent study where montelukast medicine co-administration practices were investigated in a two-stage dissolution approach using a mini-paddle apparatus (9). Similar to the results observed in this study, Martir et al. showed that mixing medicines with food and drinks can affect drug product performance, and the *in vitro* dissolution of montelukast was higher under simulated fed intestinal conditions compared to simulated fasted state conditions (9).

To further investigate the predictive value of the *in vitro* dissolution results, data could be incorporated into PBPK models, which take into account other factors that also have the potential to affect drug absorption (*i.e.* permeability, gut metabolism, *etc.*) (5). Coupling of data from *in vitro* dissolution tests with PBPK modeling is already a useful strategy used by the pharmaceutical industry during adult formulation development. PBPK models allow mechanistic understanding of drug dissolution and absorption. The optimization of both age-appropriate dissolution testing and PBPK modeling for pediatrics will be helpful to support the development of age-appropriate medicines and exploring the risk of off-label scenarios that would not be tested otherwise (45). Further studies are needed to better characterize GI physiology and its associated variability in pediatric patients. As more data becomes available on pediatric physiology, the developed dissolution set-ups could be further optimized. Future experiments with a wider range of compounds and formulations will also boost the confidence in these biopharmaceutics tools.

## CONCLUSIONS

Dissolution of montelukast from two different formulations from biorelevant dissolution set-ups that take into account changes in GI physiology as a function of age revealed that drug dissolution is significantly affected by the gastrointestinal differences between the adult and the pediatric population.

Dissolution was highly influenced by the *in vitro* dissolution apparatus (*in vitro* hydrodynamics) and by the simulation of the gastric and the intestinal conditions. The impact of both age-related gastrointestinal fluid composition and hydrodynamics can affect *in vitro* drug dissolution behaviour which can compromise drug bioavailability. These effects could be evaluated during pediatric formulation development by performing a pediatric biopharmaceutics risk assessment with the use of age-appropriate dissolution testing.

Age-related biorelevant dissolution of Singulair^®^ granules successfully predicted the *in vivo* qualitative effect of the co-administration of the formulation with food in infants. The age-related biorelevant dissolution testing proposed can support the assessment of medicine co-administration practices with food and drinks.

This study enhances our knowledge regarding the design of age-appropriate *in vitro* biopharmaceutics tools. The methods discussed should be explored, and adjusted based on knowledge of the drug and study design. The most biorelevant and clinical relevant approach will inherently depend on the drug and formulation properties, and therefore, multiple methods should be explored.

The use of *in vitro* tools like the ones described in this study can improve the understanding of the impact of age-related changes on drug dissolution and assess the influence of medicines co-administration practices, and other ‘what if’ clinical scenarios, that would be otherwise neglected.
